# Identification and verification of vascular cell adhesion protein 1 as an immune-related hub gene associated with the tubulointerstitial injury in diabetic kidney disease

**DOI:** 10.1080/21655979.2021.1976540

**Published:** 2021-09-10

**Authors:** Yan Jia, Hui Xu, Qi Yu, Lishan Tan, Zuying Xiong

**Affiliations:** a Nephrology Department, Peking University Shenzhen Hospital, Shenzhen Peking University-The Hong Kong University of Science and Technology Medical Center, Shenzhen, China; b Department of Pathology, Guangzhou Women and Children’s Medical Center, Guangzhou Medical University, Guangzhou, China; c Renal Division, Department of Medicine, Peking University First Hospital, Peking University Institute of Nephrology, Key Laboratory of Renal Disease, Ministry of Health of China, Beijing, China

**Keywords:** DKD, tubulointerstitium, immune cells, pyroptosis, VCAM1, disulfiram

## Abstract

Diabetic kidney disease (DKD) is the leading cause of chronic kidney disease (CKD) and end-stage renal disease (ESRD), but the pathogenesis is not completely understood. Tubulointerstitial injury plays critical roles in the development and progression of DKD. The present study aimed to investigate the profile of tubulointerstitial immune cell infiltration and reveal the underlying mechanisms between tubular cell injury and interstitial inflammation in DKD using bioinformatics strategies. First, xCell analysis identified immune cells displaying significant changes in the DKD tubulointerstitium, including upregulated CD4^+^ T cells, Th2 cells, CD8^+^ T cells, M1 macrophages, activated dendritic cells (DCs) and conventional DCs, as well as downregulated Tregs. Second, pyroptosis was identified as the main form of cell death compared with other forms of programmed cell death. Vascular cell adhesion protein 1 (VCAM1) was identified as the top ranked hub gene. The correlation analysis showed that VCAM1 was significantly positively correlated with pyroptosis and infiltrated immune cells in the tubulointerstitium. Upregulation of VCAM1 in the DKD tubulointerstitium was further verified in European Renal cDNA Bank cohort and was observed to negatively correlate with the glomerular filtration rate (GFR). Our *in vitro* study validated increased VCAM1 expression in HK-2 cells under diabetic conditions, and pyroptosis inhibition by disulfiram decreased VCAM1 expression, inflammatory cytokine release and fibrosis. In conclusion, our study identified upregulated VCAM1 expression in renal tubular cells, which might interact with infiltrated immune cells, thus promoting fibrosis. The FDA-approved drug disulfiram might improve fibrosis in DKD by targeting tubular pyroptosis and VCAM1 expression.

## Introduction

Diabetic kidney disease (DKD), one of the common complications of diabetes, is the main cause of chronic kidney disease (CKD) and end-stage renal disease (ESRD) in many developed and developing countries [1,2]. According to a report from the China Kidney Disease Network (CK-NET), DKD accounts for 26.70% of all cases of CKD and imposes a large medical and economic burden [2]. The pathogenesis of DKD is complex and involves a multitude of different pathways. Clarifying the pathological characteristics and pathogenesis of DKD will help improve clinical management and identify new therapeutic targets.

Although glomerular damage is the main pathological feature of DKD, increasing evidence has recently revealed that tubulointerstitial pathology [3,4], such as tubular atrophy, interstitial fibrosis and interstitial infiltrating immune cells, plays a critical role in the development and progression of DKD [5–7]. Bioinformatics analysis represents an effective method to process large amounts of data within an extremely short time and provides valuable information about disease. However, bioinformatics studies investigating tubulointerstitial gene expression and immune cell infiltration in DKD are relatively rare.

The purpose of the study was to describe the characteristics of DKD tubulointerstitial immune cell infiltration and to identify some key immune and inflammatory genes to provide novel insights into the pathogenesis and therapy of DKD. First, a tubulointerstitial microarray dataset of DKD was downloaded from the Gene Expression Omnibus (GEO) database. Using xCell [8], a web-based tool that performs a cell type enrichment analysis of gene expression data for 64 immune and stromal cell types, we first investigated the differences in immune cell infiltration between kidney tissues from individuals with DKD and normal controls. Second, a list of immune-related differentially expressed genes (DEGs) was screened, among which the top ranked gene, vascular cell adhesion protein 1 (VCAM1), was identified as an immune hub gene in DKD. Then, gene sets associated with various forms of cell death were obtained from GeneCards, and pyroptosis was evaluated as the main form of cell death using gene set variation analyses (GSVA) and gene set enrichment analysis (GSEA). Furthermore, a correlation analysis showed that VCAM1 was positively correlated with tubulointerstitial immune cell infiltration and pyroptosis. The upregulated expression of VCAM1 in the DKD tubulointerstitium was further verified in the European Renal cDNA Bank (ERCB) cohort and was observed to negatively correlate with renal function in patients with DKD. An *in vitro* study validated increased VCAM1 expression in HK-2 cells cultured under diabetic conditions, and pyroptosis inhibition by the FDA-approved drug disulfiram decreased VCAM1 expression, inflammatory cytokine release and fibrosis. Therefore, tubular pyroptosis and upregulated VCAM1 expression might be targets for immunotherapy of DKD.

## Methods

### Microarray data

The human gene expression dataset GSE30529 was downloaded from the National Center for Biotechnology Information (NCBI) Gene Expression Omnibus (GEO, http://www.ncbi.nlm.nih.gov/geo/) database. GSE30529 (GPL571 [HG-U133A_2] Affymetrix Human Genome U133A 2.0 Array) consisted of 10 DKD tubule samples and 12 control samples.

### Data processing

The raw data in the GSE30529 dataset were downloaded and processed using the Limma package and Oligo package, respectively [9,10]. Data processing included background correction, normalization, and expression calculation. When multiple probes mapped to one gene symbol, the average expression level of the probes was calculated and regarded as the gene expression level of that gene. The probes that did not map to genes were removed.

### Immune and stromal cell analyses

We applied a novel gene signature-based method, xCell [8], to estimate the cell type enrichment score and determine the profile of infiltrating immune cells in the tubulointerstitium of patients with DKD. xCell is a method for cell-type enrichment analysis using single-sample gene set enrichment analysis (ssGSEA) that calculates the enrichment scores for 64 cell types, including 34 types of immune cells, 30 types of stromal cells and other cells. It outperforms other extensive in silico analyses (including CIBERSORT) by encompassing more types of immune cells and employing a spillover compensation technique to reduce dependencies between closely related cell types. The 34 types of immune cells were categorized into nine groups, including CD4^+^ T cell subpopulations, CD8^+^ T cell subpopulations, gamma delta T cells (Tγδ cells), NK cells, NKT cells, B cell subpopulations, monocyte/macrophage subpopulations, dendritic cell (DC) subpopulations, and granulocyte subpopulations.

### Gene set enrichment analyses (GSEA) and gene set variation analyses (GSVA)

Using the hallmark gene sets as the reference gene set, we performed gene set variation analyses (GSVA) between DKD and control tissues using the GSVA Bioconductor package **[**11**]**. The thresholds were set to enrichment score change > 1.0, p value < 0.05 and t value >2. Both upregulated and downregulated pathways were identified. GSEA were performed with the GSEA desktop application **[**12**]**, and a false discovery rate (FDR) <0.25 and p value <0.05 were set as the thresholds.

### Construction of gene sets representing various forms of cell death and enrichment analysis

Gene sets representing various forms of cell death were constructed by searching the keywords ‘pyroptosis’, ‘necrosis’, ‘necroptosis’, ‘apoptosis’, ‘ferroptosis’ and ‘autophagy’ in the GeneCards database (https://www.genecards.org/). Nonprotein-coding genes were removed. The gene sets are listed in Table S2. Then, gene set GSEA were performed using the GSEA desktop application to evaluate the cell death form in the tubulointerstitium. We performed Pearson’s correlation analyses to explore the relationship between pivotal cell death pathways and infiltrating immune cells in the tubulointerstitium.

### Analysis of DEGs and screening of immune hub genes

DEGs between patients with DKD and healthy controls were identified using the ‘Limma’ package **[**13**]** in R software. An adjusted P value<0.05 and log fold change (FC) ≥ 1 were set as the thresholds. A list of 1793 immune-related genes was downloaded from ImmPort Shared Data (https://www.immport.org/shared/genelists), and shared genes from the two datasets (DEGs and immune-relevant gene list) were determined by constructing a Venn diagram to identify immune-related hub genes. An analysis of the functional interactions between proteins might provide insights into the mechanisms of DKD. In the present study, the protein–protein interaction (PPI) networks of ninety-four shared genes were generated using the STRING database (https://string-db.org/) and visualized with Cytoscape software (version 3.7.1, http://www.cytoscape.org/). An interaction score greater than 0.4 (medium confidence) was considered statistically significant. CytoHubba, a Cytoscape plugin, was utilized to explore PPI network hub genes **[**14**]**. Among them, VCAM1 was the top ranked gene. Enrichment analyses of the thirty immune hub genes identified above were performed using the online tool Metascape **[**15**]**. Furthermore, dominant modules in the PPI network were identified using the MCODE plugin (version 1.4.2, http://apps.cytoscape.org/apps/MCODE) in Cytoscape software.

### VCAM1 verification and correlation analysis with renal function

The differential expression of VCAM1 in the tubulointerstitium of patients with DKD was validated in the ERCB cohort (31 healthy controls and 17 patients with DKD). Then, we used the Nephroseq v5 online database (http://v5.nephroseq.org), an integrated data-mining platform for gene expression datasets of kidney diseases, to validate the correlation between VCAM1 expression and clinical traits of patients with DKD using Pearson’s correlation analysis in ERCB. A p value of <0.05 was considered statistically significant.

### Immunofluorescence staining for VCAM1

Paraffin kidney sections from a patient clinicopathologically diagnosed with DKD were stained with VCAM1. This was approved by the Committee on Research Ethics of Peking University First Hospital (NO. 20171280). Nonspecific binding was blocked with 3% BSA following fixation and heat treatment for antigen retrieval. Kidney sections were then incubated with a primary antibody against VCAM1 (Abcam, ab134047, 1:200) overnight at 4°C. The Cy3-labeled secondary antibody was obtained from Jackson ImmunoResearch. Tumor-adjacent kidney tissue from nephrectomy samples was used as a normal control. Representative images were captured using a Leica DFC 7000 T camera via Leica Application Suite V4.7.1 software.

### Cell culture

Human kidney 2 (HK-2) cells, a human kidney proximal tubular epithelial cell line, were cultured in DMEM (11885084, Gibco, USA) supplemented with 10% fetal bovine serum (10099141 C, Gibco, USA) and 1% penicillin/streptomycin (10378016, Gibco, USA) at 37°C with 5% CO_2_. High glucose (30 mM D-glucose, G8644, Sigma) **[**16**]** and TNF-α (40 ng/ml, 300–01A, PeproTech) **[**17**]** were used to induce HK-2 cell injury *in vitro*. Disulfiram (DSL, 0.3 μM **[**18,19**]**, NSC190940, Selleck), a pore-formation inhibitor, was added 3 hours prior to the induction of HK-2 cell injury.

### Western blot

Total protein was extracted from HK-2 cells using RIPA buffer (Sigma, R0278), and the protein concentration was determined using a Pierce BCA Protein Assay kit (Thermo Fisher Scientific, 23227). Next, denatured proteins were separated by sodium dodecyl sulfate-polyacrylamide gel electrophoresis (SDS–PAGE) and then electrically transferred to polyvinylidene difluoride membranes (Millipore, IPVH00010). The membranes were blocked for 60 minutes with 5% fat-free milk dissolved in Tris-buffered saline containing 0.1% Tween 20 (TBST). The blots were incubated with the following relevant primary antibodies overnight at 4°C: GSDMD (Abcam, ab209845, 1:1000), VCAM1 (Abcam, ab134047, 1:2000), and tubulin (ZSBio, TA-10, 1:5000). An incubation with a 1:1000 dilution of the HRP-conjugated secondary antibody was carried out for 1 hour at room temperature. After five washes with TBST, the membranes were incubated with the chemiluminescence substrate (Millipore, WBKLS0100) for 5 minutes, and images were captured using an Image Quant LAS 4000 Mini system (GE Healthcare). The semiquantitative analysis was conducted using ImageJ software (Media Cybernetics, Silver Spring, MD).

### RNA isolation and RT–PCR analysis

Total RNA was extracted from HK-2 cells using an RNAsimple Total RNA Kit (Tiangen, DP419) according to the manufacturer’s instructions and reverse transcribed into cDNAs using a FastKing RT Kit (Tiangen, KR116). Quantative PCR was performed using a StepOne Real-Time PCR System (Applied Biosystems, USA) with two-step methods. The sequences of the primers used are shown in Table S4. Comparative gene expression was calculated using the 2^−ΔΔ*Ct*^ method as described previously.

### Statistical analysis

GraphPad Prism 6.0 software was used for statistical analyses, and data are presented as the means ± SEM. A two-tailed unpaired t test was applied for comparisons between two groups. Differences at the P < 0.05 level were considered statistically significant.

## Results

Accumulating evidence has recently revealed that tubulointerstitial pathology plays a critical role in the development and progression of DKD. The purpose of the study was to describe the characteristics of DKD tubulointerstitial immune cell infiltration and to identify some key immune and inflammatory genes to provide novel insights into the pathogenesis and therapy of DKD. In this study, we investigated the profile of tubulointerstitial immune cell infiltration and identified pyroptosis as the main form of programmed cell death in DKD using bioinformatics analysis. VCAM1 was identified as the top ranked immune-related hub gene and was positively correlated with pyroptosis and infiltrated immune cells. Furthermore, VCAM1 expression was validated to be elevated in renal tubular cells cultured under diabetic conditions. The FDA-approved drug disulfiram inhibited renal tubular cell pyroptosis and decreased VCAM1 expression, inflammatory cytokine levels and fibrosis *in vitro*.

### 1. Bioinformatics analysis workflows

**Figure 1.**
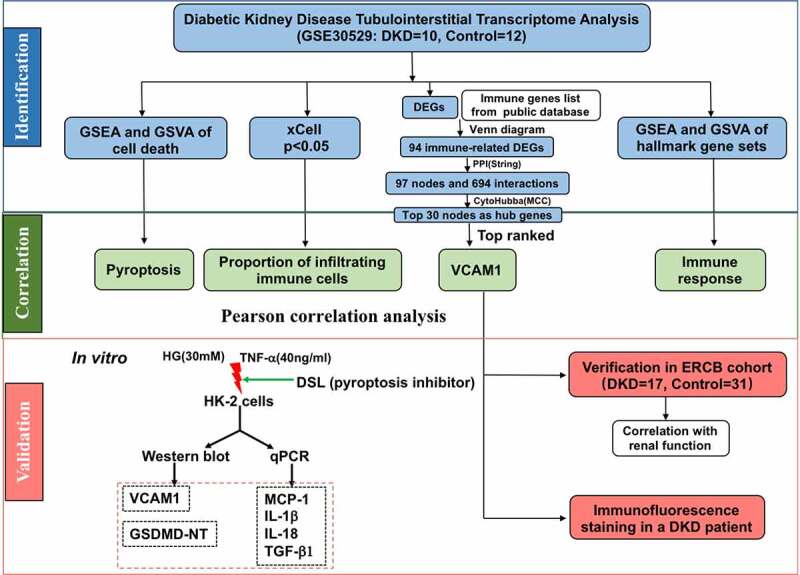
Workflow of this study

The main steps of the workflow are shown in Figure 1. We first evaluated immune cell infiltration in tubulointerstitial tissues from patients with DKD. Then, pyroptosis, the main form of cell death in the tubulointerstitium, was discovered using GSEA and GSVA. Next, we screened a list of genes closely related to immune cell infiltration and identified hub genes. A correlation analysis was performed between infiltrated immune cells, pyroptosis and the top hub gene VCAM1. In addition, clinical validation and immunostaining validation of VCAM1 were performed. Finally, we further conducted an *in vitro* study to validate the results of the bioinformatics analysis.

### 2. Immune and stromal cell deconvolution analyses

**Figure 2.**
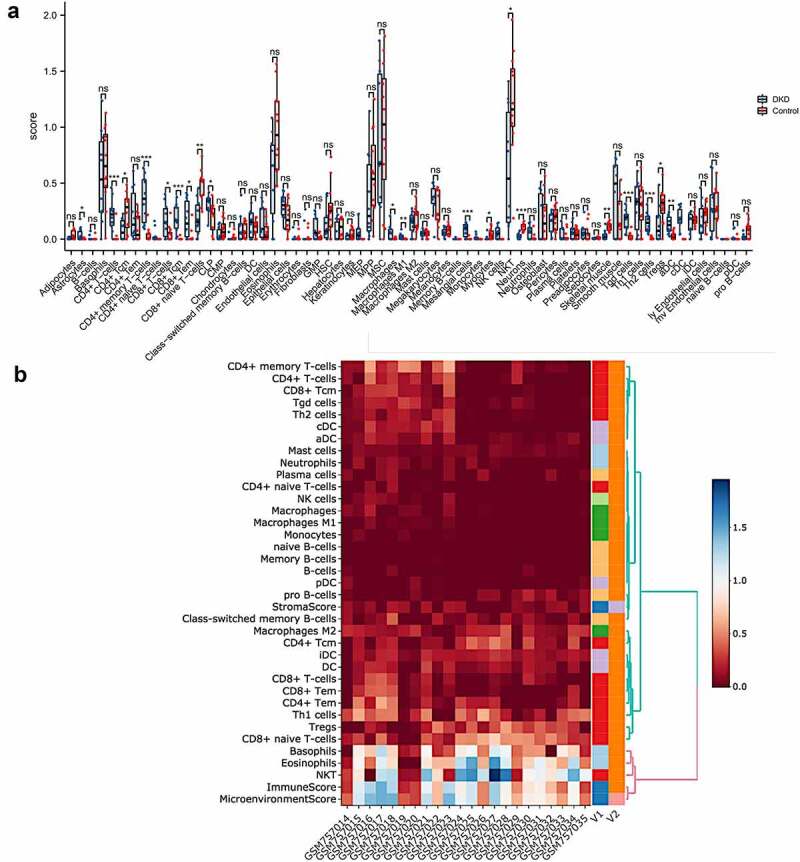
**Immune cell infiltration analysis by xCell in the tubulointerstitium of DKD**. (a) Comparison of xCell scores of 64 cell types in the tubulointerstitium between DKD and control renal tissue in the GSE30529 dataset. (b) The cellular landscape of immune microenvironment in DKD. The heatmap represents cell type enrichment score of each immune cell type for all samples. DC, dendritic cell; Tcm, central memory T cell; Tem, effective memory T cell; Tγδ cell, gamma delta T cell

We used xCell, which generates cell type enrichment scores using bulk gene expression data, to determine the cell types potentially involved in tubulointerstitial injury in diabetic kidney disease. The enrichment scores of 64 cell types, including 34 types of immune cells and 30 types of stromal and other cells, were obtained for each sample (Figure 2(a)). The heatmap of 34 immune cell enrichment scores was illustrated to identify the immune landscape of the DKD tubulointerstitium (Figure 2(b)). Compared with normal controls, the majority of T cell enrichment scores were relatively higher in patients with DKD, except for CD4^+^ Tcm, Tregs, Th1 cells, CD8^+^ naïve T cells, and NKT cells. The enrichment scores of aDCs and cDCs were higher in patients with DKD than in controls, while Pro-B cell enrichment scores were lower in patients with DKD. The enrichment scores of most monocyte/macrophage and granulocyte subsets were not significantly different between DKD and normal tissues, except for M1 macrophages and neutrophils and eosinophils (Table 1).

**Table 1.** Immune cell enrichment score between DKD patients and normal controls

	DKD (n = 10) Mean±SEM	Controls (n = 12) Mean±SEM	*P*
CD4 T-cell subpopulations			
CD4 + T cells	0.2182 ± 0.04533	0.02152 ± 0.01897	**0.0004**
CD4+ naïve T cells	0.03083 ± 0.01899	0.0 ± 0.0	0.089
CD4+ memory T cells	0.3547 ± 0.06656	0.03815 ± 0.01823	**< 0.0001**
CD4+ Tcm	0.08278 ± 0.02926	0.2400 ± 0.04904	**0.0166**
CD4+ Tem	0.2146 ± 0.07198	0.1041 ± 0.03488	0.1605
Tregs	0.1340 ± 0.05184	0.3015 ± 0.04379	**0.0218**
Th1 cells	0.2988 ± 0.07070	0.2918 ± 0.05794	0.9388
Th2 cells	0.1490 ± 0.02815	0.00575 ± 0.003196	**< 0.0001**
CD8 T-cell subpopulations			
CD8 + T cells	0.1662 ± 0.04715	0.04317 ± 0.02055	**0.0195**
CD8+ naïve T cells	0.1898 ± 0.05078	0.4839 ± 0.04030	**0.0002**
CD8+ Tcm	0.1845 ± 0.03968	0.00305 ± 0.00305	**< 0.0001**
CD8+ Tem	0.1787 ± 0.05322	0.02509 ± 0.01853	**0.0083**
Tγδ cells	0.1690 ± 0.02973	0.02208 ± 0.009720	**< 0.0001**
NKT	0.6282 ± 0.1711	1.212 ± 0.1347	**0.0132**
NK cells	0.05611 ± 0.01876	0.0 ± 0.0	**0.0036**
B-cell subpopulations			
Pro B cells	0.01628 ± 0.008225	0.08178 ± 0.02194	**0.0174**
B cells	0.01107 ± 0.007845	0.004783 ± 0.004783	0.4861
Naïve B cells	0	0	na
Memory B cells	0.00178 ± 0.00178	0.0 ± 0.0	0.284
Class-switched memory B cells	0.09659 ± 0.03978	0.07359 ± 0.01983	0.5918
Plasma cells	0.04556 ± 0.01902	0.0240 ± 0.01357	0.3563
Monocyte/macrophage subpopulations			
Monocytes	0.0131 ± 0.007538	0.0 ± 0.0	0.0701
Macrophages	0.05801 ± 0.02097	0.003867 ± 0.002350	**0.0107**
Macrophages M1	0.02475 ± 0.008952	0.0006083 ± 0.0006083	**0.0078**
Macrophages M2	0.1425 ± 0.03050	0.1866 ± 0.03174	0.3341
DC subpopulations			
DCs	0.1409 ± 0.03783	0.1027 ± 0.01948	0.3565
Activated DCs	0.1304 ± 0.02466	0.02792 ± 0.009929	**0.0005**
Conventional DCs	0.1838 ± 0.03091	0.0 ± 0.0	**< 0.0001**
Plasmacytoid DCs	0.02108 ± 0.01302	0.003875 ± 0.003875	0.1862
Immature DCs	0.1495 ± 0.02799	0.1745 ± 0.01547	0.423
Granulocyte subpopulations			
Neutrophils	0.06308 ± 0.01922	0.01414 ± 0.01003	**0.028**
Eosinophils	0.5600 ± 0.1245	0.9532 ± 0.1106	**0.0281**
Mast cells	0.05088 ± 0.009363	0.04652 ± 0.008655	0.7361
Basophils	0.5578 ± 0.1299	0.6611 ± 0.09912	0.5276
NOTE: Bold indicates statistical significance.			
Abbreviations: DC, dendritic cell; Tem, effector memory T cell; Tγδ cell, gamma delta T cell.

### 3. GSVA and GSEA of hallmark gene sets

**Figure 3.**
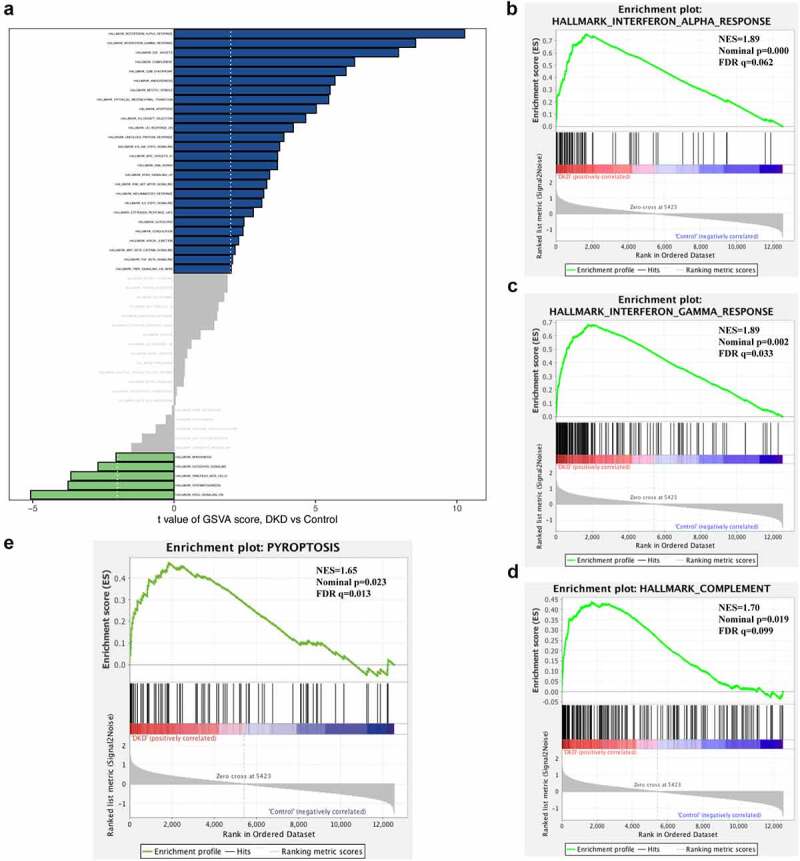
**Gene set enrichment analyses (GSEA) and gene set variation analyses (GSVA)**. (a) Barplot of GSVA results of 50 hallmark gene sets. (b)-(d) GSEA results of ‘Hallmark_ interferon alpha response’, ‘Hallmark_ interferon gamma response’ and ‘Hallmak complement’. (e) GSEA result of pyroptosis

We conducted GSVA and GSEA of hallmark gene sets in the GSE30529 dataset to explore the biological processes in the DKD tubulointerstitium. The ‘interferon alpha response’, ‘interferon gamma response’ and ‘complement’ signaling pathways were significantly activated in the pathogenesis of tubulointerstitial injury in patients with DKD (Figure 3(a)), and a summary of the GSVA results is displayed in Table S1. Then, we performed GSEA of the three gene sets and found that they were positively enriched in the dataset. The normalized enrichment scores (NESs) were 1.89, 1.89 and 1.70, with normalized p values (NOM p values) of 0.000, 0.002 and 0.019, respectively (Figure 3(b-d)). All of the significantly enriched pathways identified using GSEA are shown in Table 2. These data suggest that the tubulointerstitial immune response plays important roles in the pathogenesis of DKD.

**Table 2.** Significantly enriched hallmark gene sets using GSEA

GS follow link to MSigDB	GS DETAILS	NES	NOM p-val	FDR q-val	LEADING EDGE
**1**	HALLMARK INTERFERON ALPHA RESPONSE	1.89	0.000	0.062	tags = 71%, list = 13%, signal = 81%
**2**	HALLMARK INTERFERON GAMMA RESPONSE	1.89	0.002	0.033	tags = 61%, list = 14%, signal = 70%
**3**	HALLMARK ANGIOGENESIS	1.81	0.006	0.048	tags = 37%, list = 8%, signal = 40%
**4**	HALLMARK E2F TARGETS	1.71	0.006	0.105	tags = 48%, list = 21%, signal = 60%
**5**	HALLMARK COMPLEMENT	1.70	0.019	0.099	tags = 35%, list = 13%, signal = 39%
**6**	HALLMARK EPITHELIA MESENCHYMAL TRANSITION	1.66	0.046	0.103	tags = 43%, list = 14%, signal = 49%
**7**	HALLMARK G2M CHECKPOINT	1.61	0.028	0.140	tags = 42%, list = 19%, signal = 51%
**8**	HALLMARK ALLOGRAFT REJECTION	1.55	0.115	0.196	tags = 43%, list = 14%, signal = 49%
**9**	HALLMARK APOPTOSIS	1.54	0.020	0.184	tags = 31%, list = 14%, signal = 35%
**10**	HALLMARK MITOTIC SPINDLE	1.52	0.037	0.187	tags = 30%, list = 16%, signal = 35%
**11**	HALLMARK UV RESPONSE DN	1.48	0.044	0.225	tags = 46%, list = 22%, signal = 58%

### 4. GSEA and GSVA of gene sets associated with various forms of cell death

Cell death includes pyroptosis, necrosis, necroptosis, apoptosis, ferroptosis and autophagy, which have different pathophysiological mechanisms and signaling pathways. Various forms of programmed cell death have been reported to be associated with inflammatory and immune responses. We constructed gene sets of several types of cell death from GeneCards (Table S2) and then performed both GSEA and GSVA to explore the involvement of cell death in tubulointerstitial inflammation in patients with DKD. GSEA showed that only the pyroptosis gene sets were significantly enriched in patients with DKD compared to normal controls (NES = 1.65, FDR q value = 0.023, FWER = 0.013) (Figure 3(e)). Meanwhile, the GSVA score for pyroptosis was the highest among all of the various types of cell death analyzed (Table S3). Based on this result, pyroptosis might play a role in the pathogenesis of tubulointerstitial injury in patients with DKD.

### 5. Correlation between pyroptosis enrichment scores and immune cell infiltration

**Figure 4.**
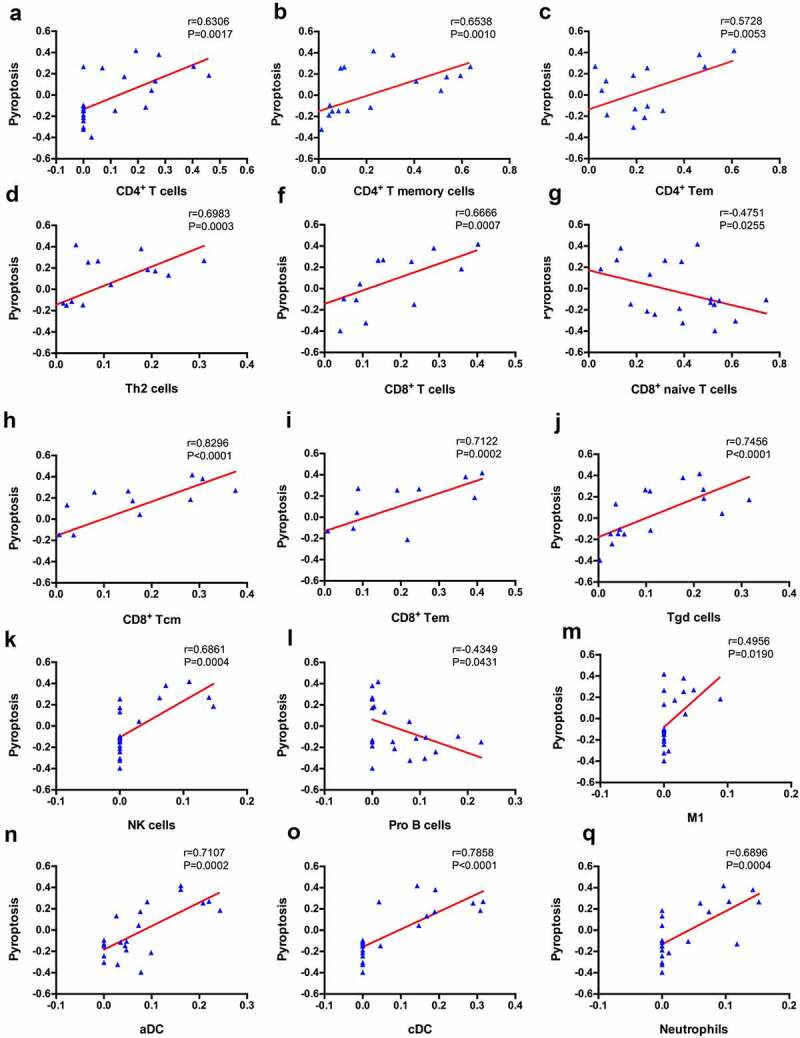
Correlation between immune cells and pyroptosis

GSVA enrichment scores for pyroptosis were correlated with the proportion of immune cell infiltration by performing Pearson’s correlation analysis to evaluate the correlation between pyroptosis and infiltration of immune cells. We observed a significant correlation between 15 types of immune cells and pyroptosis (Figure 4), suggesting a close interaction between immune cells and pyroptosis.

### 6. Discovery of core genes

**Figure 5.**
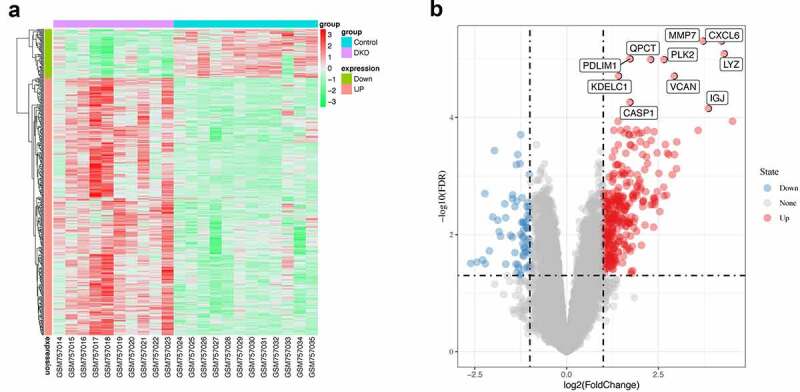
**Differentially expressed genes**. (a) Heatmap of potential differentially expressed genes in the tubulointerstitium between DKD and control renal tissue. (b) Volcano plot of potential differentially expressed genes in the tubulointerstitium between DKD and control renal tissue. The top ten differentially genes are shown

**Figure 6.**
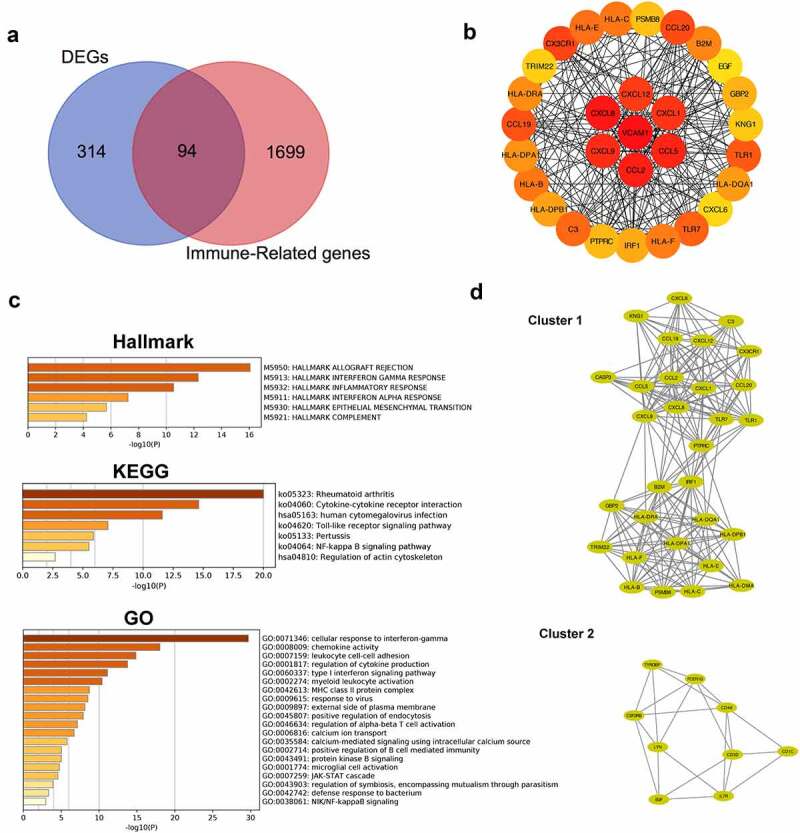
**Identification of hub genes and enrichment analysis**. (a) Venn diagram of DEGs and immune-related gene list. (b) Top thirty hub immune genes identified via PPI network. (c) Hallmark, KEGG and GO enrichment analysis of the top thirty hub immune genes. (d) Two most significant clusters of DEGs were identified by MCODE plugin

A differential expression analysis was performed to further explore the relationship between renal tubular cells and interstitial immune cells. Four hundred eight DEGs were identified between the DKD group and the control group, among which 65 were downregulated and 343 upregulated (Figure 5). An immune-related gene list was downloaded to identify immune-related DEGs, and ninety-four hub immune genes were acquired from two datasets (DEGs and the immune-related gene list) via a Venn diagram (Figure 6(a)). The PPI analysis of these ninety-four hub immune genes revealed 97 nodes and 694 interactions (Figure S1). The top 30 nodes calculated by the MCC algorithm were identified as hub genes (Figure 6(b) and Table 3). Then, an enrichment analysis of hub genes was performed. ‘Allograft rejection’, ‘interferon gamma response’, ‘inflammatory response’, ‘interferon alpha response’, ‘epithelial mesenchymal transition’ and ‘complement’ were the most enriched items in the hallmark analysis. ‘Rheumatoid arthritis’, ‘cytokine-cytokine receptor interaction’, ‘Toll-like receptor signaling pathway’, ‘pertussis’ and ‘NF-kappa B signaling pathway’ were the most enriched KEGG terms. ‘Cellular response interferon gamma’, ‘chemokine activity’, ‘leukocyte cell-cell adhesion’, and ‘regulation of cytokine production’ were the most enriched GO terms (Figure 6(c)). Furthermore, the MCODE scoring system identified two clusters with a score ≥5. PTRPC, TRIM22, PSMB8, KNG1, and CXCL6 were hub nodes with higher node degrees in module 1 (Figure 6(d)), and CD1C, EGF, FCER1G, CD3D and CD48 were hub nodes in module 2 (Figure 6(e)).

**Table 3.** Top 30 in network string_interactions.tsv ranked by MCC method

Rank	Name	Score	Rank	Name	Score
1	VCAM1	2.41E+09	15	HLA-B	9.63E+08
2	CXCL8	1.46E+09	17	HLA-F	9.63E+08
3	CCL2	1.46E+09	18	B2M	9.63E+08
4	CCL5	1.46E+09	19	HLA-DRA	9.62E+08
5	CXCL9	1.45E+09	20	HLA-DPA1	9.62E+08
6	CXCL1	1.45E+09	21	HLA-DQA1	9.62E+08
7	CXCL12	1.45E+09	22	HLA-DPB1	9.62E+08
8	CX3CR1	1.45E+09	23	IRF1	9.60E+08
9	CCL20	1.44E+09	24	GBP2	9.59E+08
10	CCL19	1.44E+09	25	PTPRC	4.97E+08
11	TLR1	9.80E+08	26	PSMB8	4.83E+08
12	TLR7	9.80E+08	27	KNG1	4.80E+08
13	C3	9.64E+08	28	TRIM22	4.79E+08
14	HLA-E	9.63E+08	29	CXCL6	4.79E+08
15	HLA-C	9.63E+08	30	EGF	1.21E+07

### 7. Correlation between VCAM1 expression and infiltrating immune cells

**Figure 7.**
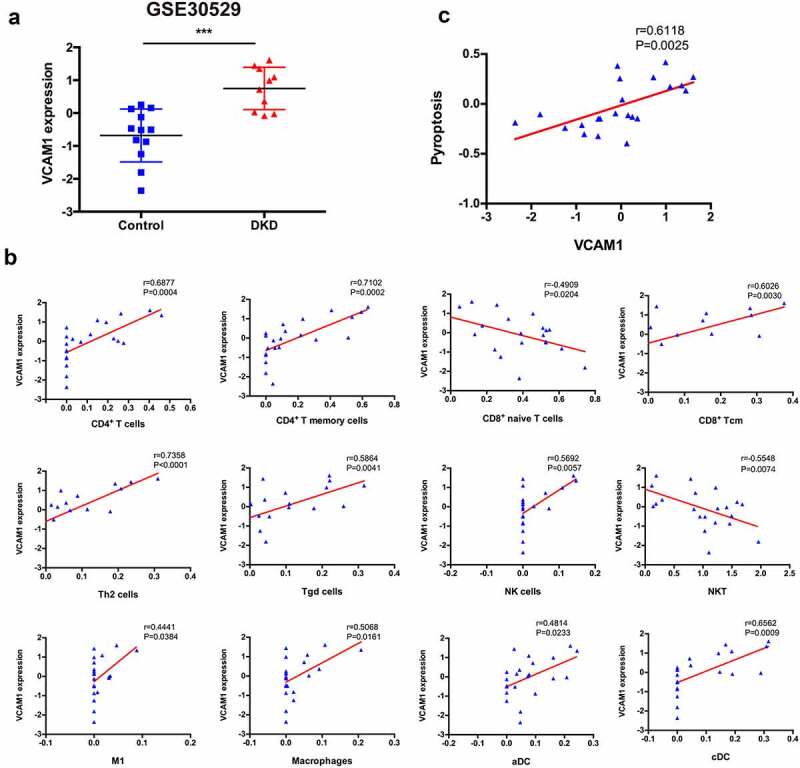
**Correlation between VCAM1 and immune cells and pyroptosis**. (a) VCAM1 expression was significantly increased in patients with DKD (n = 10) compared with healthy controls (n = 12) (***P < 0.001). (b) Correlation of VCAM1 with the infiltrated immune cells. (c) Correlation of VCAM1 with pyroptosis

Among the hub genes identified, the top ranked gene, VCAM1, was identified as an immune hub gene in DKD. VCAM1 expression was significantly increased in patients with DKD (Figure 7(a)). The correlation analysis revealed that VCAM1 expression was positively correlated with CD4^+^ T cells (r = 0.6877, P = 0.0004), CD4^+^ T memory cells (r = 0.7102, P = 0.0002), Th2 cells (r = 0.7358, P < 0.0001), CD8^+^ Tcm (r = 0.6026, P = 0.0030), Tγδ cells (r = 0.5864, P = 0.0041), NK cells (r = 0.5692, P = 0.0057), macrophages (r = 0.5068, P = 0.0161), M1 macrophages (r = 0.4441, P = 0.0384), aDCs (r = 0.4814, P = 0.0233) and cDCs (r = 0.6562, P = 0.0009) but negatively correlated with naïve CD8^+^ T cells (r = −0.4909, P = 0.0204) and NKT cells (r = −0.5548, P = 0.0074) (Figure 7(b)).

### 8. Correlation between VCAM1 expression and pyroptosis

Pearson’s correlation analysis was performed to explore whether the upregulated VCAM1 expression in the tubulointerstitium of patients with DKD is related to pyroptosis. VCAM1 expression was closely related to pyroptosis (r = 0.6118, P = 0.0025) (Figure 7(c)), indicating that pyroptosis might induce VCAM1 expression.

### 9. Verification, clinical relevance and immunofluorescence validation of VCAM1 expression

**Figure 8.**
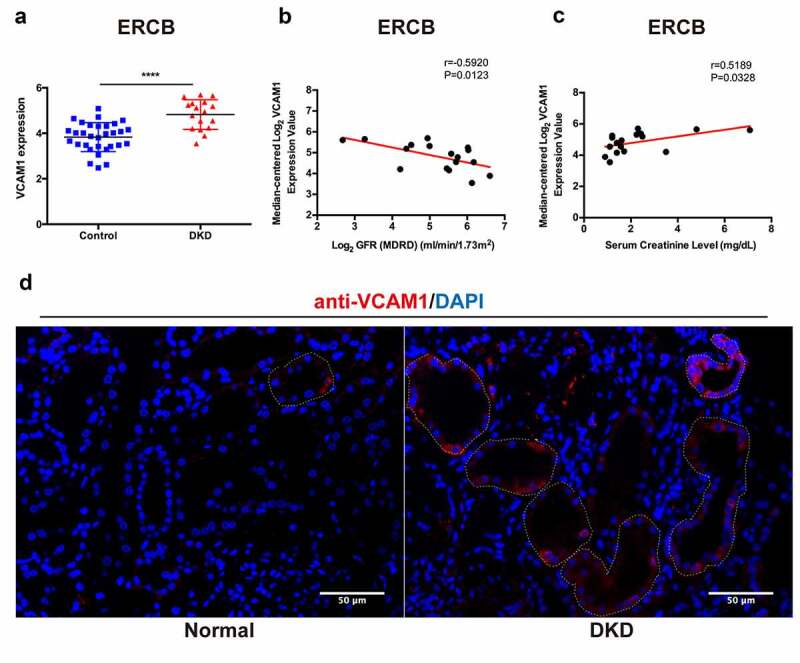
**Verification, clinical relevance and immunofluorescence validation of VCAM1**. (a) Verification of the increased VCAM1 expression in DKD in ERCB cohort (31 healthy controls and 17 DKD) (****P < 0.0001). (b) Correlation between VCAM1 expression and GFR in ERCB. (c) Correlation between VCAM1 expression and serum creatinine in ERCB. (d) Immunofluorescence staining of VCAM1 in normal kidney tissue and DKD. The tubular basements are outlined. Scale bars, 50 μm

We performed a relevant analysis of patients with DKD from ERCB to validate the change in VCAM1 expression in the DKD tubulointerstitium. Consistently, VCAM1 mRNA levels were significantly higher in patients with DKD (Figure 8(a)). VCAM1 expression was negatively correlated with the glomerular filtration rate (GFR) (r = −0.5920, P = 0.0123) (Figure 8(b)) and positively correlated with serum creatinine levels (r = 0.5189, P = 0.0328) (Figure 8(c)). Furthermore, we performed immunofluorescence staining for VCAM1 in the kidney tissue of a patient with DKD and found that renal tubular VCAM1 expression was significantly upregulated compared with that in normal kidneys (Figure 8(d)).

### 10. DSL inhibited HG-induced HK-2 cell pyroptosis, VCAM1 expression and inflammation

**Figure 9.**
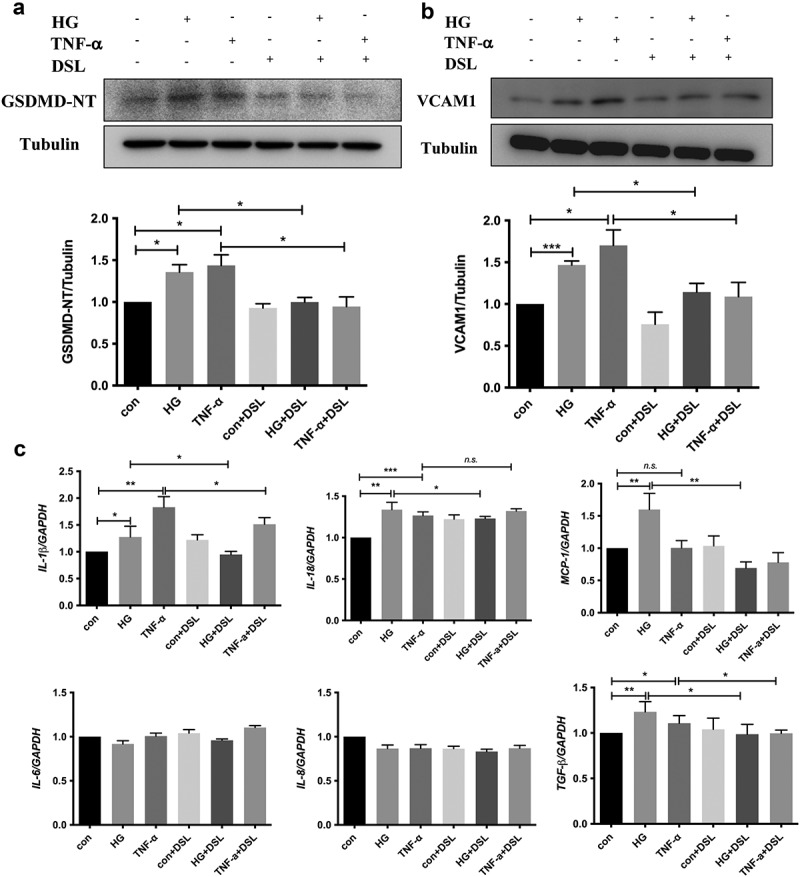
**DSL inhibited HG-induced HK-2 cell pyroptosis, VCAM1 expression and inflammation**. (a) Western blot detected the expression of GSDMD-NT and VCAM1. n = 4. (b) Quantitative reverse-transcription PCR of IL-1β, IL-18, MCP-1, TGF-β1 in HK-2 cells. n = 4. Data are means ± SEM. *P < 0.05, **P < 0.01, ***P < 0.001

We exposed cultured HK-2 cells to high glucose (HG) and TNF-α for 48 h to further determine the relationship between tubular cell pyroptosis, VCAM1 expression and the tubulointerstitial immune response in individuals with DKD. Since the core of cellular pyroptosis is membrane pore formation induced by cleaved GSDMD, we detected the levels of the GSDMD N-terminal domain (GSDMD-NT) and VCAM1 using western blotting. Both HG and TNF-α induced a significant increase in the GSDMD-NT level, indicating the occurrence of GSDMD-mediated pyroptosis (Figure 9(a)). VCAM1 expression was increased statistically significantly in the HG and TNF-α groups (Figure 9(b)). Disulfiram (DSL) is an effective inhibitor of GSDMD pore formation [18]. Pretreatment with DSL alleviated the increase in GSDMD-NT levels, indicating that cell pyroptosis was suppressed. Meanwhile, VCAM1 expression was decreased in the DSL-pretreated HG and TNF-α groups (Figure 9(a-b)). Next, the inflammatory cytokine profile was determined using qPCR. The results showed that the upregulated levels of the *MCP-1, IL-1β, and IL-18* mRNAs induced by HG were significantly decreased after DSL pretreatment. In the TNF-α group, the level of *IL-1β* decreased significantly in the DSL-pretreated group, and the levels of *MCP-1* and *IL-18* did not change significantly. No significant changes in the levels of the *IL-6* and *IL-8* mRNAs were found in any group. Importantly, *TGF-β1* mRNA levels were also decreased in the DSL-pretreated groups. Thus, pyroptotic tubular cells increased VCAM1 expression under diabetic conditions and that a treatment abrogating pyroptosis inhibited VCAM1 expression, inflammation and fibrosis in HK-2 cells.

## Discussion

Currently available clinical approaches to treating DKD remain limited and mainly involve strict control of hyperglycemia, proteinuria and blockade of the renin-angiotensin system. Considering the high incidence of DKD-related ESRD, novel and satisfactory strategies are urgently needed. Accumulating evidence indicates a paramount role for immunity and inflammation in the pathogenesis of DKD. To date, several studies have screened genes related to glomerular immune cell infiltration in individuals with DKD [20]. Relevant tubulointerstitial studies are rare. In the present study, we analyzed immune cell infiltration and relevant cellular processes in the tubulointerstitium using bioinformatics analysis. Pyroptosis was identified as the main form of cell death among other forms of programmed cell death using GSEA and GSVA. VCAM1 was identified as the top immune-related hub gene and was correlated with renal function in patients with DKD. An *in vitro* study validated increased VCAM1 expression in HK-2 cells cultured under diabetic conditions, and pyroptosis inhibition by disulfiram decreased VCAM1 expression, inflammatory cytokine release and fibrosis. Our study identified a close relationship between renal proximal tubular pyroptosis, VCAM1 expression and the interstitial immune response in patients with DKD. This study provided new insights into the tubulointerstitial pathogenesis of DKD and helped identify new therapeutic targets.

xCell was used to conduct a comprehensive evaluation of immune cell infiltration in individuals with DKD. The results revealed that both innate immunity and adaptive immunity were involved in tubulointerstitial injury in patients with DKD. Macrophages and DCs constitute the mononuclear phagocyte (MNP) system **[**21**]**, which is particularly complex in the kidney **[**22**]**. We found that macrophages, especially M1 macrophages, were significantly enriched in the tubulointerstitium. Interstitial macrophage M1 infiltration was reported to be correlated with interstitial fibrosis, tubular atrophy, renal function and proteinuria in both animal models and human kidneys **[**23–25**]**. M1 macrophages produce cytokines and proteins (e.g., Nitric oxide, platelet derived growth factor, IL-1, and TNF-α), causing damage to vascular endothelial cells and the proliferation of fibroblasts and mesangial cells, thus aggravating interstitial fibrosis **[**26**]**. DCs were reported to be involved in tubulointerstitial inflammation in various progressive kidney diseases, such as lupus nephritis **[**27,28**]** and crescentic glomerulonephritis **[**29,30**]**. Nevertheless, the role of DCs in many prevalent kidney diseases, such as DKD and hypertensive nephropathy, remains poorly understood. Only one animal study showed that CD11^+^ dendritic cells infiltrated the glomeruli in NOD mice and correlated with albuminuria **[**31**]**, and no dendritic cell infiltration in the interstitium has been reported until now. The vast majority of kidney DCs are conventional DCs (cDCs) expressing CD11b and C-X3-C motif receptor 1 **[**32,33**]**. We observed increased infiltration of DCs, especially activated DCs and conventional DCs, indicating a role for these cells in the tubulointerstitial pathogenesis of DKD. Previous studies have reported that DCs were identified morphologically within the tubulointerstitium and might be involved in the pathogenesis of interstitial inflammation in drug-induced acute interstitial nephritis **[**34**]** and light chain-associated tubulointerstitial nephritis **[**35**]**. The specific mechanism requires additional experimental evidence. Helper (CD4^+^) T cells, cytotoxic (CD8^+^) cells and B cells are important components of the adaptive immune system. In the present study, we observed a significant enrichment of CD4^+^ and CD8^+^ T cells in the interstitium, suggesting a role of adaptive immunity in DKD. An animal study also detected CD4^+^ and CD8^+^ T cell infiltration in the kidney interstitium in streptozotocin-treated rats **[**36**]**. In people with diabetes, the number of CD4^+^ T cells and CD20 cells positively correlates with proteinuria **[**37**]**. However, not all CD4^+^ T cells promote DKD. In our study, Tregs were downregulated in DKD and almost negatively correlated with pyroptosis (r = −0.4191, P = 0.0522). Tregs appear to play a protective role in DKD.

Renal tubular epithelial cells are critical for both tubular and glomerular function, and injured renal tubular cells are an important source of proinflammatory cytokines and chemokines **[**38**]**. Multiple studies have shown that regulated cell death (RCD), such as pyroptosis, necroptosis, apoptosis, and ferroptosis, of tubular cells contributes to the pathophysiology of kidney diseases **[**39–42**]**. Various forms of cell death were evaluated using GSEA and GSVA in the dataset to explore the relationship between tubular injury and interstitial immune cell infiltration. The results revealed that pyroptosis was the main form of cell death compared with necroptosis, apoptosis, ferroptosis and autophagy. The accumulation of reactive oxygen species (ROS) in individuals with DKD caused by hyperglycemia is an important activator of the NLRP3 (NOD-like receptor family, pyrin domain containing 3) inflammasome. Then, NLRP3 activates gasdermin D (GSDMD) and rapidly causes cell membrane rupture and the release of cell contents, leading to an inflammatory response **[**43**]**. Pyroptosis is involved in many chronic progressive diseases, such as Alzheimer’s disease **[**44**]**, Parkinson’s disease **[**45**]**, atherosclerosis **[**46**]** and rheumatoid arthritis **[**47**]**. The correlation analysis indicated that pyroptosis was positively correlated with increased interstitial infiltration of CD4^+^ T cells, CD4^+^ T memory cells, Th2 cells, CD8^+^ T cells, CD8^+^ Tcm cells, CD8^+^ Tem cells, Tγδ cells, NK cells, M1 macrophages, activated dendritic cells, conventional dendritic cells and neutrophils. Thus, our study showed that tubular pyroptosis-related interstitial inflammation might be an important contributor to DKD. This finding was confirmed in studies of patients with diabetes and db/db mice showing that tubular epithelial cell pyroptosis was accompanied by increases in the levels of the NLPR3 inflammasome, IL-1β, and TGF-β. Treatments targeting renal tubular epithelial cell pyroptosis may be critical to interfere with the progression of DKD.

**Figure 10.**
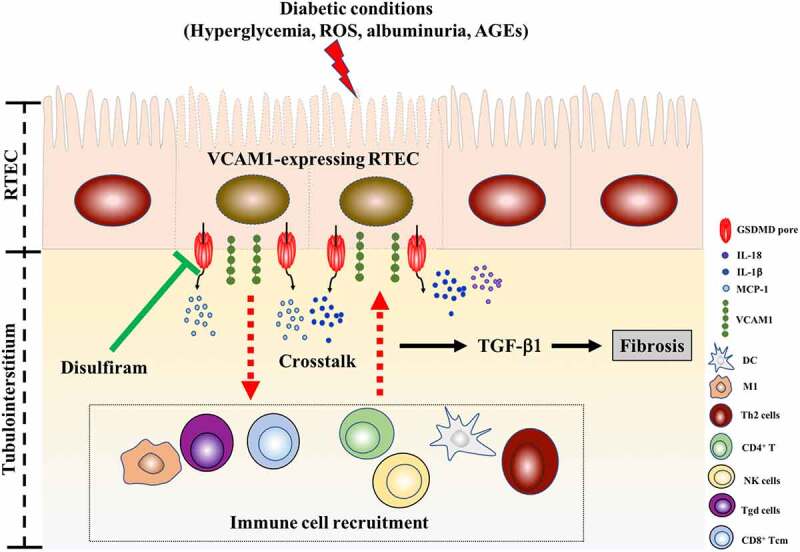
A proposed model of VCAM1-expressing tubular epithelial cell promoting interstitial inflammation and fibrosis in DKD, and their inhibition by disulfiram

The highest MCC score for VCAM1 obtained from cytoHubba suggested that it is an important hub gene in DKD tubulointerstitial injury. VCAM1 has an important role in leukocyte adhesion to the endothelium. According to an animal study, MRL/lpl (a murine model of lupus nephritis) kidneys display increased VCAM1 expression in the endothelium, cortical tubules and glomeruli **[**48**]**. Patients with type 2 diabetes present significantly elevated serum levels of VCAM1, and VCAM1 levels correlate well with the extent of albuminuria **[**49**]**. Previous studies on VCAM1 in DKD mainly mainly focused on glomerular endothelial cells **[**50**]**. However, researchers have not completely elucidated whether renal tubular expression of VCAM1 is elevated in patients with DKD and whether it is involved in the interstitial pathogenesis of DKD. In the present study, the upregulated VCAM1 mRNA levels in the DKD tubulointerstitium in the public GEO dataset and markedly increased tubular VCAM1 expression in HK-2 cells confirmed the upregulation of tubular VCAM1 expression in DKD. As VCAM1 is an adhesion molecule for lymphocytes and monocytes, the positive correlation between VCAM1 expression and interstitial immune cell infiltration in our study indicated that elevated tubular VCAM1 expression might allow the protein to interact with mononuclear cells, thus promoting interstitial fibrosis. Consistent with our results, an increase in tubular VCAM1 expression in episodes of acute renal allograft rejection is associated with T cell and monocyte infiltration **[**50**]**. In addition, we found a positive correlation between VCAM1 expression and pyroptosis, indicating that pyroptotic tubular cells might release VCAM1. The *in vitro* study validated increased VCAM1 expression in HK-2 cells cultured under diabetic conditions, and pyroptosis inhibition by disulfiram decreased VCAM1 expression, inflammatory cytokine release and fibrosis. Our study highlighted renal tubular VCAM1 as an attractive target for DKD therapy (Figure 10). However, the molecular mechanisms underlying the interaction between tubular VCAM1 and immune cell infiltration in the interstitium are essentially unknown, and further experimental studies are urgently needed **[**51**]**.

The current work investigated the profile of tubulointerstitial immune cell infiltration and identified pyroptosis as the main form of programmed cell death in patients with DKD using bioinformatics analysis. The analytical methods were reliable and novel. The correlation between immune cells, pyroptosis, and VCAM1 provides new information for future research. Furthermore, our *in vitro* study suggested that the FDA-approved drug disulfiram might alleviate interstitial inflammation and fibrosis by inhibiting tubular pyroptosis and VCAM1 expression in individuals with DKD. However, this study has several limitations. First, the sample size applied in the analysis was small. Second, the results were not confirmed by conducting in-depth experiments. Thus, more combined samples will be needed, and the results will be verified by experiments in DKD animal models and DKD cohorts.

## Conclusion

In conclusion, using bioinformatics analysis, we investigated the profile of tubulointerstitial immune cell infiltration and evaluated the role of pyroptosis in the tubulointerstitium of DKD. VCAM1 was identified as the hub gene and was positively correlated with pyroptosis and infiltrated immune cells. In addition, VCAM1 expression was validated to be elevated in renal tubular cells. *In vitro* study revealed that the FDA-approved drug disulfiram inhibited renal tubular cell pyroptosis and decreased VCAM1 expression, inflammatory cytokine levels and fibrosis. Further experimental analyses of pyroptosis and VCAM1 in the tubulointerstitium of patients with DKD might identify targets for immunotherapy.

## Supplementary Material

Supplemental Material

## Availability of Data and Matierals

The datasets used during the study are available from the corresponding author upon reasonable request.
