# Supplementary material for: Identification and verification of vascular cell adhesion protein 1 as an immune-related hub gene associated with the tubulointerstitial injury in diabetic kidney disease
Source: Bioengineered. 2021 Sep 10;12(1):6655–73. doi: 10.1080/21655979.2021.1976540 (PMC8806788; doi:10.1080/21655979.2021.1976540)
Supplement: Supplemental Material [file KBIE_A_1976540_SM3843.zip › supplementary/TableS1_Summary of GSVA for hallmark gene sets.docx]

**Table S1** Summary of GSVA for hallmark gene sets

| **Tag** | **logFC** | **AveExpr** | **t** | **P.Value** | **adj.P.Val** | **B** |
| --- | --- | --- | --- | --- | --- | --- |
| HALLMARK_INTERFERON_ALPHA_RESPONSE | 0.741549016 | -0.034034391 | 10.27413153 | 7.49E-11 | 3.74E-09 | 14.78354143 |
| HALLMARK_INTERFERON_GAMMA_RESPONSE | 0.657580621 | -0.036939159 | 8.542799098 | 3.56E-09 | 8.91E-08 | 10.8939045 |
| HALLMARK_E2F_TARGETS | 0.464736592 | -0.019907187 | 7.933254839 | 1.52E-08 | 2.54E-07 | 9.429562524 |
| HALLMARK_COMPLEMENT | 0.354971271 | -0.008919799 | 6.380420395 | 7.63E-07 | 9.54E-06 | 5.493501871 |
| HALLMARK_G2M_CHECKPOINT | 0.360485445 | -0.023007138 | 6.081574083 | 1.67E-06 | 1.67E-05 | 4.707750274 |
| HALLMARK_ANGIOGENESIS | 0.374510241 | -0.014099244 | 5.688775605 | 4.74E-06 | 3.95E-05 | 3.665506625 |
| HALLMARK_MITOTIC_SPINDLE | 0.309512851 | -0.019044147 | 5.517617675 | 7.48E-06 | 5.34E-05 | 3.20889274 |
| HALLMARK_EPITHELIAL_MESENCHYMAL_TRANSITION | 0.467944726 | -0.022740654 | 5.467149812 | 8.56E-06 | 5.35E-05 | 3.074054586 |
| HALLMARK_KRAS_SIGNALING_DN | -0.400566686 | -0.002958543 | -5.073071447 | 2.47E-05 | 0.000137164 | 2.01959922 |
| HALLMARK_APOPTOSIS | 0.330982994 | -0.006278843 | 5.028662935 | 2.78E-05 | 0.00013913 | 1.900750905 |
| HALLMARK_ALLOGRAFT_REJECTION | 0.454419829 | -0.053100487 | 4.657461485 | 7.56E-05 | 0.000343811 | 0.909409433 |
| HALLMARK_UV_RESPONSE_DN | 0.340767252 | -0.003440122 | 4.214219236 | 0.000248469 | 0.001035288 | -0.262787728 |
| HALLMARK_UNFOLDED_PROTEIN_RESPONSE | 0.311187469 | 0.003630946 | 3.886345989 | 0.00059336 | 0.002282153 | -1.114592684 |
| HALLMARK_SPERMATOGENESIS | -0.237641852 | 0.005847978 | -3.749729309 | 0.000849621 | 0.002951714 | -1.46401478 |
| HALLMARK_IL6_JAK_STAT3_SIGNALING | 0.333559499 | -0.026773082 | 3.733916967 | 0.000885514 | 0.002951714 | -1.504211703 |
| HALLMARK_MYC_TARGETS_V1 | 0.399672609 | 0.019710425 | 3.663931837 | 0.001062992 | 0.003048526 | -1.681464526 |
| HALLMARK_DNA_REPAIR | 0.273209756 | 0.001441877 | 3.657559076 | 0.00108078 | 0.003048526 | -1.697550078 |
| HALLMARK_PANCREAS_BETA_CELLS | -0.271161283 | -0.004546828 | -3.651672075 | 0.001097469 | 0.003048526 | -1.712401175 |
| HALLMARK_KRAS_SIGNALING_UP | 0.248272387 | -0.015272439 | 3.377962719 | 0.002222253 | 0.005848034 | -2.393248916 |
| HALLMARK_PI3K_AKT_MTOR_SIGNALING | 0.195322446 | -0.014365947 | 3.275492629 | 0.002882591 | 0.007206478 | -2.642738669 |
| HALLMARK_INFLAMMATORY_RESPONSE | 0.298550759 | -0.031631676 | 3.171444736 | 0.003744652 | 0.008915838 | -2.892657588 |
| HALLMARK_IL2_STAT5_SIGNALING | 0.207987722 | -0.009973114 | 3.102368366 | 0.00444824 | 0.010109637 | -3.056543618 |
| HALLMARK_ESTROGEN_RESPONSE_LATE | 0.157455706 | -0.007178914 | 2.801954323 | 0.009257386 | 0.020124751 | -3.748201592 |
| HALLMARK_HEDGEHOG_SIGNALING | -0.190984879 | -0.011354083 | -2.69100008 | 0.012046878 | 0.025097663 | -3.994027654 |
| HALLMARK_GLYCOLYSIS | 0.153690847 | -0.00101407 | 2.470041611 | 0.020083587 | 0.040167174 | -4.465997489 |
| HALLMARK_COAGULATION | 0.164780621 | 0.002258096 | 2.437996257 | 0.021594435 | 0.04152776 | -4.532365725 |
| HALLMARK_APICAL_JUNCTION | 0.184792049 | -0.03092065 | 2.279481298 | 0.030717712 | 0.056884651 | -4.852318131 |
| HALLMARK_WNT_BETA_CATENIN_SIGNALING | 0.184944323 | -0.031892325 | 2.158287333 | 0.039913695 | 0.071274455 | -5.087069504 |
| HALLMARK_TGF_BETA_SIGNALING | 0.202094587 | -0.009666744 | 2.064424944 | 0.048655695 | 0.082843066 | -5.262638268 |
| HALLMARK_MYOGENESIS | -0.149815398 | -0.0042741 | -2.054181194 | 0.04970584 | 0.082843066 | -5.281457749 |
| HALLMARK_TNFA_SIGNALING_VIA_NFKB | 0.225489825 | -0.002468398 | 2.022006127 | 0.053135889 | 0.085703047 | -5.340122866 |
| HALLMARK_MTORC1_SIGNALING | 0.192733862 | 0.005461407 | 1.873348701 | 0.071828603 | 0.108927439 | -5.602142657 |
| HALLMARK_PROTEIN_SECRETION | 0.267984099 | 0.024189967 | 1.872904287 | 0.07189211 | 0.108927439 | -5.602903139 |
| HALLMARK_P53_PATHWAY | 0.109694405 | 0.0097596 | 1.760679551 | 0.089560003 | 0.131705886 | -5.79042834 |
| HALLMARK_MYC_TARGETS_V2 | 0.112514294 | 0.005228025 | 1.553136334 | 0.131976629 | 0.188538041 | -6.112542936 |
| HALLMARK_ANDROGEN_RESPONSE | 0.158535011 | -0.004505038 | 1.505530125 | 0.143740045 | 0.198407139 | -6.181706389 |
| HALLMARK_XENOBIOTIC_METABOLISM | -0.150818323 | 0.021117531 | -1.493585197 | 0.146821283 | 0.198407139 | -6.198775146 |
| HALLMARK_ESTROGEN_RESPONSE_EARLY | 0.083108346 | -0.006419043 | 1.404748597 | 0.171439873 | 0.22557878 | -6.322071857 |
| HALLMARK_BILE_ACID_METABOLISM | -0.106048662 | 0.025938514 | -1.119642472 | 0.272678527 | 0.349587855 | -6.672619887 |
| HALLMARK_HYPOXIA | 0.055395374 | 0.00579674 | 0.920909733 | 0.365212408 | 0.45651551 | -6.874089298 |
| HALLMARK_OXIDATIVE_PHOSPHORYLATION | -0.093539789 | 0.045848954 | -0.633652835 | 0.531612354 | 0.648307749 | -7.099366346 |
| HALLMARK_UV_RESPONSE_UP | 0.046130215 | 0.007813011 | 0.597804744 | 0.554926737 | 0.660627068 | -7.121835519 |
| HALLMARK_APICAL_SURFACE | 0.038559336 | -0.0156869 | 0.448349546 | 0.657460405 | 0.764488843 | -7.201708832 |
| HALLMARK_PEROXISOME | 0.039935108 | 0.007811979 | 0.365618462 | 0.717484412 | 0.80129318 | -7.236251296 |
| HALLMARK_REACTIVE_OXYGEN_SPECIES_PATHWAY | 0.033295609 | 0.00511722 | 0.360635817 | 0.721163862 | 0.80129318 | -7.238109926 |
| HALLMARK_NOTCH_SIGNALING | 0.025328344 | 0.004252265 | 0.323089443 | 0.74910399 | 0.814243467 | -7.251302461 |
| HALLMARK_ADIPOGENESIS | -0.033510755 | 0.03027375 | -0.277839299 | 0.783241595 | 0.83323574 | -7.265290114 |
| HALLMARK_CHOLESTEROL_HOMEOSTASIS | 0.006396392 | 0.0042594 | 0.078300667 | 0.938163404 | 0.967730685 | -7.301911739 |
| HALLMARK_HEME_METABOLISM | -0.004711247 | 0.026706906 | -0.061615684 | 0.951320462 | 0.967730685 | -7.303116424 |
| HALLMARK_FATTY_ACID_METABOLISM | 0.005358542 | 0.03584269 | 0.040829572 | 0.967730685 | 0.967730685 | -7.304215379 |
