# Supplementary material for: Identification and verification of vascular cell adhesion protein 1 as an immune-related hub gene associated with the tubulointerstitial injury in diabetic kidney disease
Source: Bioengineered. 2021 Sep 10;12(1):6655–73. doi: 10.1080/21655979.2021.1976540 (PMC8806788; doi:10.1080/21655979.2021.1976540)
Supplement: Supplemental Material [file KBIE_A_1976540_SM3843.zip › supplementary/TableS3_Summary of GSVA for gene sets of cell death.docx]

**Table S3** Summary of GSVA for gene sets of cell death

| Tag | logFC | AveExpr | t | P.Value | adj.P.Val | B |
| --- | --- | --- | --- | --- | --- | --- |
| Pyroptosis | 0.398257 | -0.018865 | 7.279549 | 2.78E-07 | 1.39E-06 | 6.8845499 |
| Necrosis | 0.130051 | -0.004649 | 5.736782 | 9.15E-06 | 1.68E-05 | 3.39917 |
| Necroptosis | 0.298749 | -0.005856 | 5.695630 | 1.01E-05 | 1.68E-05 | 3.3025515 |
| Apoptosis | 0.133833 | -0.001136 | 4.943149 | 6.10E-05 | 7.63E-05 | 1.5132755 |
| Autophagy | 0.224822 | -0.000383 | 4.387900 | 2.29E-04 | 2.74E-04 | 0.2004597 |
| Ferroptosis | 0.182998 | 0.014687 | 1.641324 | 0.114997 | 0.114997 | -5.641412 |
