# Supplementary material for: Identification and verification of vascular cell adhesion protein 1 as an immune-related hub gene associated with the tubulointerstitial injury in diabetic kidney disease
Source: Bioengineered. 2021 Sep 10;12(1):6655–73. doi: 10.1080/21655979.2021.1976540 (PMC8806788; doi:10.1080/21655979.2021.1976540)
Supplement: Supplemental Material [file KBIE_A_1976540_SM3843.zip › supplementary/TableS4_primers.docx]

| **Table S4 Primers used for real-time PCR** | | |
| --- | --- | --- |
| **Genes** | **Forward primers (5'-3')** | **Reverse primers (5'-3')** |
| MCP-1 | CCTTCATTCCCCAAGGGCTC | GGTTTGCTTGTCCAGGTGGT |
| IL-6 | CACTGGTCTTTTGGAGTTTGAG | GGACTTTTGTACTCATCTGCAC |
| IL-8 | AACTGAGAGTGATTGAGAGTGG | ATGAATTCTCAGCCCTCTTCAA |
| IL-1β | GAGCTCGCCAGTGAAATGATG | GTGGTGGTCGGAGATTCGTA |
| IL-18 | TCTTCATTGACCAAGGAAATCGG | TCCGGGGTGCATTATCTCTAC |
| TGF-β | CTGTACATTGACTTCCGCAAG | TGTCCAGGCTCCAAATGTAG |
| GAPDH | CTCTGCTCCTCCTGTTCGAC | GCGCCCAATACGACCAAATC |
|  | | |
