# Supplementary figures and images for: Identification and verification of vascular cell adhesion protein 1 as an immune-related hub gene associated with the tubulointerstitial injury in diabetic kidney disease
Source: Bioengineered. 2021 Sep 10;12(1):6655–73. doi: 10.1080/21655979.2021.1976540 (PMC8806788; doi:10.1080/21655979.2021.1976540)

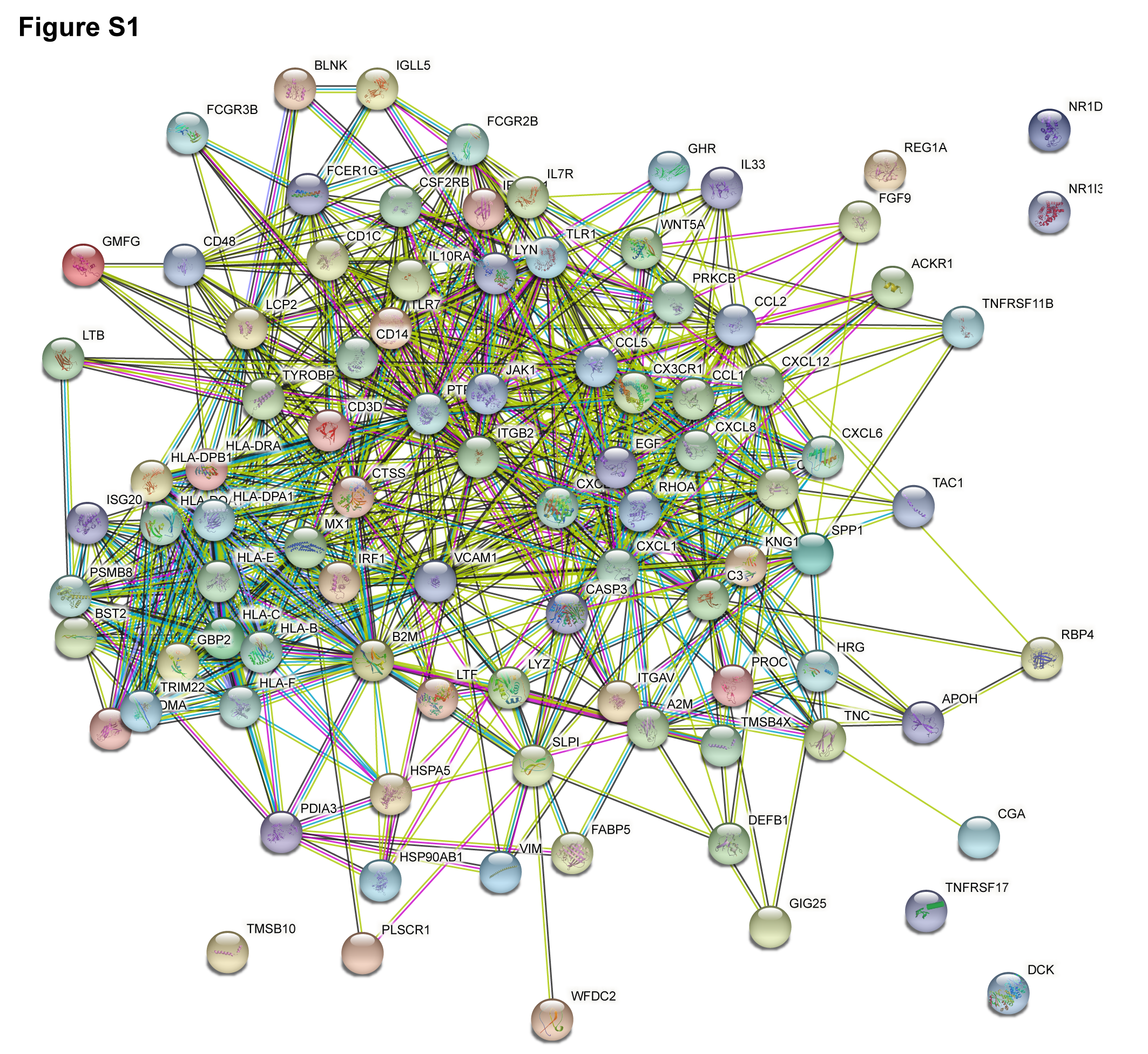

Supplement: Supplemental Material [file KBIE_A_1976540_SM3843.zip › supplementary/Figure_S1.tif]
